# Response Surface Optimized Extraction of 1-Deoxynojirimycin from Mulberry Leaves (*Morus alba* L.) and Preparative Separation with Resins

**DOI:** 10.3390/molecules19067040

**Published:** 2014-05-30

**Authors:** Teng Wang, Cui-Qing Li, Hong Zhang, Ji-Wen Li

**Affiliations:** 1 Department of Pharmaceutical Engineering, Beijing Institute of Petro-chemical Technology, Beijing 102617, China; 2 Department of Chemical Engineering, Beijing Institute of Petro-chemical Technology, Beijing 102617, China; 3 College of Science, Agricultural University of Hebei, Baoding 071001, China

**Keywords:** 1-deoxynojirimycin, extraction, response surface methodology, resin

## Abstract

In the present study, the extraction technology and preparative separation of 1-deoxynojirimycin from mulberry leaves were systematically investigated. Four extraction parameters (ethanol concentration, extraction temperature, extraction time and ratio of solvent to sample) were explored by response surface methodology (RSM). The results indicated that the maximal yield of 1-deoxynojirimycin was achieved with an ethanol concentration of 55%, extraction temperature of 80 °C, extraction time of 1.2 h and ratio of solvent to sample of 12:1. The extraction yield under these optimum conditions was found to be 256 mg/100 g dry mulberry leaves. A column packed with a selected resin was used to perform dynamic adsorption and desorption tests to optimize the separation process. The results show that the preparative separation of 1-deoxynojirimycin from mulberry leaves can be easily and effectively done by adopting 732 resin. In conclusion, 732 resin is the most appropriate for the separation of 1-deoxynojirimycin from other components in mulberry leaves extracts, and its adsorption behavior can be described with Langmuir isotherms and a two-step adsorption kinetics model. The recovery and purity of 1-deoxynojirimycin in the final product were 90.51% and 15.3%, respectively.

## 1. Introduction

Mulberry leaves (*Morus alba* L.), cultivated mainly in Asian countries, have drawn the interest of many researchers because they were found to have antihyperglycemic, antioxidant and antimicrobial activities [1,2,3]. Mulberry leaves are rich in flavonoid, alkaloid and polysaccharide components which are known to be the major bioactive compounds from chemical constituent investigations. Among those, 1-deoxynojirimycin (1-DNJ), which is known as a potent α-glycosidase inhibitor, has gained considerable attention for use as a functional or medical additive to control blood glucose [4,5]. These pharmaceutical discoveries led to interest in the exploration of effective extraction and preparative separations of 1-DNJ from mulberry leaves.

In order to prepare bioactive compounds, solvent extraction was often used and the extraction yield is significantly influenced by many factors, such as solvent concentration, extraction temperature, extraction time, and ratio of solvent to sample, *etc.* [6,7,8]. The traditional optimization method was single-factor experiments, in which only one factor is varied at a time while others are fixed at constant values. However, this method is time-consuming and cannot evaluate the interaction effects among the various factors. Response surface methodology (RSM) is an effective technique to overcome these problems [9]. It can explore the relationships between the response values and the independent variables and optimize the processes where multiple variables may influence the outputs [10]. Up to now, RSM has been successfully used to model and optimize the biochemical and biotechnological processes related to food systems [11,12,13].

The future use of 1-DNJ is bright, but the amount of the key constituent in mulberry leaves is as low as only 0.1% [14,15], hence, a more efficient and simple purification method for 1-DNJ is required. The purification method with resin is a new promising technology, and has been successfully applied in the preparative separation of natural products [16]. Similarly, such a method is extremely useful for the extraction and separation of the 1-DNJ from mulberry leaves.

The objective of the present study was to investigate the influence of ethanol concentration, extraction temperature, extraction time and ratio of solvent to sample on the yield of 1-DNJ using RSM. Furthermore, the adsorption and desorption properties of 1-DNJ from mulberry leaves with different resins were investigated and an efficient method for the preparative separation of 1-DNJ from mulberry leaves with 732 resin has been developed.

## 2. Results and Discussion

### 2.1. Optimization of Extraction Parameters of 1-DNJ

#### 2.1.1. Fitting the Response Surface Model

The effect of four independent variables (ethanol concentration (*X*_1_), extraction temperature (*X*_2_), extraction time (*X*_3_) and ratio of solvent to sample (*X*_4_)) on the yield of extraction (*Y*) was investigated using a four-factor and three levels Box-Behnken design (Table 1).

**Table 1:** Experimental design using Box-Behnken and the extraction yields of 1-DNJ.

Number	*X*_1_	*X*_2_	*X*_3_	*X*_4_	*Y* (mg/100g dry powder)
1	0	0	−1	−1	224
2	0	0	−1	1	240.8
3	0	−1	0	−1	174.8
4	0	0	1	−1	248.8
5	0	1	1	0	160.4
6	1	1	0	0	125.2
7	−1	−1	0	0	105.2
8	−1	1	0	0	154
9	0	0	0	0	249.2
10	1	0	0	1	110.8
11	1	0	1	0	175.2
12	0	0	0	0	244.4
13	0	0	0	0	246
14	−1	0	0	1	250.8
15	1	0	0	−1	149.2
16	0	1	0	−1	128.8
17	0	1	−1	0	127.2
18	−1	0	0	−1	114.8
19	1	−1	0	0	168.8
20	0	0	0	0	245.6
21	−1	0	−1	0	172.8
22	0	1	0	1	164.4
23	1	0	−1	0	114.4
24	0	−1	−1	0	212.8
25	0	0	1	1	246.4
26	−1	0	1	0	104.4
27	0	−1	1	0	158.8
28	0	−1	0	1	148.8
29	0	0	0	0	249.2

Multiple linear regression analysis was performed based on the results in Table 1 using the following second-order polynomial Equation (1):

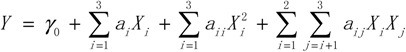
(1)
where *Y* is the predicted response, *γ*_0_ is a constant, *a_i_*, *a_ii_* and *a_ij_* are respectively the linear, quadratic and interactive coefficients of the model. Accordingly, *X_i_* and *X_j_* represent the levels of the independent variables, respectively. The response variable and the independent variables are related by the following second-order polynomial equation [Equation (2)]:

*Y* = 246.88 − 4.86*X*_1_− 9.1*X*_2_ + 0.17*X*_3_ + 10.13*X*_4_− 23.11*X*_1_*X*_2_ + 32.3*X*_1_*X*_3_− 43.6X_1_X_4_ + 21.8*X*_2_*X*_3_ + 15.4*X*_2_*X*_4_− 4.8*X*_3_*X*_4_− 71.14*X*_1_^2^− 60.69*X*_2_^2^− 16.09*X*_3_^2^− 14.04*X*_4_^2^ (2)

The statistics test of the model was performed by “analysis of variance” (ANOVA) and the results are shown in Table 2. The value of the determination coefficient (*R*^2^) of the regression model was 0.8432, which indicated that 84.32% of the variations could be explained by the fitted model, suggesting that a closely correlation was achieved as the *R*^2^ value was higher than 0.8 [17]. The “Adequate Precision” measures the signal to noise ratio and a ratio greater than 4 is desirable. In this study, the ratio was found to be 7.835, indicating that this model can be used to navigate the design space [18].

The significance of the regression model was checked using the *P*-value and the model would be more significant with a smaller *P*-value. The ANOVA of the quadratic regression model demonstrated that the model was highly significant, as was evident from a very low probability value (*p* = 0.001). Furthermore, the significance of the model was also determined by “Lack of Fit” test, the *F*-value of the “Lack of Fit” was 259.88, which suggested that it was not significant and only a 0.01% chance could occur due to noise [19].

Additionally, the *p*-value was also used to evaluate the significance of each coefficient, which might indicate the pattern of the interactions between the variables. In this case, the cross product coefficients (*X*_1_*X*_3_, *X*_1_*X*_4_) and the quadratic term coefficients (*X*_1_^2^, *X*_2_^2^) were significant with a very small *p*-value (*p* < 0.05), while the other term coefficients were not significant (*p* > 0.05).

**Table 2:** The regression coefficients and ANOVA results.

Source	DF	Sum of Square	Mean Square	*F*-value	*p*-value
Model	14	67,638.64	4831.33	5.3781	0.0017
*X*_1_	1	284.21	284.21	0.3163	0.5827
*X*_2_	1	993.72	993.72	1.1061	0.3107
*X*_3_	1	0.33	0.333	0.0004	0.9849
*X*_4_	1	1232.21	1232.21	1.3716	0.2611
*X*_1_*X*_2_	1	2134.44	2134.44	2.3760	0.1455
*X*_1_*X*_3_	1	4173.16	4173.16	4.6455	0.0490
*X*_1_*X*_4_	1	7603.84	7603.84	8.4645	0.0114
*X*_2_*X*_3_	1	1900.96	1900.96	2.1161	0.1678
*X*_2_*X*_4_	1	948.64	948.64	1.0560	0.3216
*X*_3_*X*_4_	1	92.16	92.16	0.1026	0.7535
*X*_1_^2^	1	32,827.46	32,827.45	36.5431	<0.0001
*X*_2_^2^	1	23,891.52	23,891.5	26.5957	0.0001
*X*_3_^2^	1	1679.27	1679.27	1.8693	0.1931
*X*_4_^2^	1	1278.62	1278.62	1.4233	0.2527
Lack of Fit		-	-	259.88	0.0001
Pure Error	4	19.28	4.83		
Total	28	80,215.13			

*R*^2^ = 0.8432, Adequate Precision = 7.835.

#### 2.1.2. Analysis of the Response Surface

**Figure 1:**
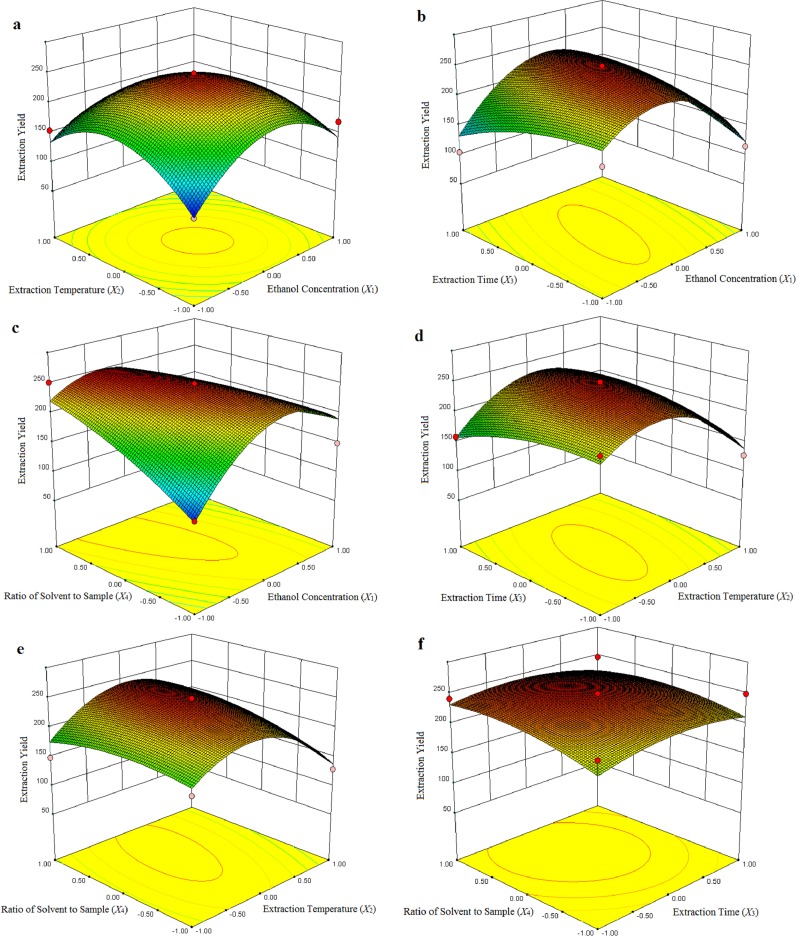
Response surface plots showing the effect of ethanol concentration (*X*_1_), extraction temperature (*X*_2_), extraction time (*X*_3_) and ratio of solvent to sample (*X*_4_) on the yield of 1-DNJ.

3D response surface plots provide a method to visualize the relationship between responses and experimental levels of each variable and the type of interactions between two test variables. Different shapes of the contour plots indicated different interactions between the variables. Circular contour plot indicated that the interactions between the corresponding variables were negligible, while elliptical contour plot indicated that the interactions between the corresponding variables were significant [20,21]. As shown in Figure 1a, when extraction time (*X*_3_) and ratio of solvent to sample (*X*_4_) were kept at zero level, ethanol concentration (*X*_1_) and extraction temperature (*X*_2_) were showed reciprocal interaction on the extraction yield. When extraction temperature (*X*_2_) kept at lower level, the yield of 1-DNJ increased at first, and then decreased with the increase of ethanol concentration (*X*_1_). The circular contour plot indicated that the interactions between ethanol concentration (*X*_1_) and extraction temperature (*X*_2_) were insignificant. Likewise, the interaction of the extraction time (*X*_3_) and ratio of solvent to sample (*X*_4_) were demonstrated insignificantly on the extraction yield of 1-DNJ from Figure 1f. Figure 1b shows the influence of ethanol concentration (*X*_1_) and extraction time (*X*_3_) demonstrating quadratic effects on the yield of 1-DNJ when the other two variables were fixed at zero level. The mutual interaction between ethanol concentration (*X*_1_) and extraction time (*X*_3_) was significant which may be concluded by the type of the contour plot.

Figure 1c shows the response surface at varying ethanol concentration (*X*_1_) and ratio of solvent to sample (*X*_4_) with the fixed extraction temperature (zero level) and extraction time (zero level). It can be concluded that the extraction yield of 1-DNJ was affected significantly by ethanol concentration (*X*_1_) and ratio of solvent to sample (*X*_4_). The extraction yield increased with the increase of ratio of solvent to sample (*X*_4_) when ethanol concentration (*X*_1_) was kept at lower level, while decreased with the increase of ratio of solvent to sample (*X*_4_) when ethanol concentration (*X*_1_) was kept at higher level.

Figure 1d shows the interactive influences of the extraction temperature (*X*_2_) and extraction time (*X*_3_) while the other two variables were kept at zero level. The yield of 1-DNJ was slightly decreased with the increase of extraction time (*X*_3_) when extraction temperature (*X*_2_) was kept the lower level. Furthermore, it can be concluded that the interactive influence between extraction temperature (*X*_2_) and extraction time (*X*_3_) was insignificant. Likewise, Figure 1e showed that the interaction of extraction temperature (*X*_2_) and ratio of solvent to sample (*X*_4_) were demonstrated insignificantly on the extraction yield of 1-DNJ. The extraction yield of 1-DNJ increased slightly with the increase of ratio of solvent to sample (*X*_4_) when extraction temperature (*X*_2_) was kept at lower level.

#### 2.1.3. Validation of the Model Equation

The suitability of the model for predicting the optimum response values was tested by using the optimal conditions with small modifications. The maximum predicted yield and experimental yield of 1-DNJ were presented in Table 3. The experiments by using the predicted optimum extraction conditions for 1-DNJ were as follows: ethanol concentration of 55.2%, extraction temperature of 80.4 °C, extraction time of 1.2 h, ratio of solvent to sample of 12:1, and the model predicted a maximum yield of 256 mg/100 g dry powder. In order to ensure the predicted result, validation experiment was performed by using the modified conditions: the concentration of ethanol of 55%, extraction temperature of 80 °C, extraction time of 1.2 h, ratio of solvent to sample of 12:1. A mean value of 255 ± 2.54 mg/100 g dry powder was obtained from validation experiments. The good correlation among these results confirmed that the response model was adequate for reflecting the expected optimization. The results of analysis indicated that the experimental values were in agreement with the predicted one, and also suggested that the model was satisfactory and accurate.

**Table 3:** Predicted and experimental values of the response under the optimal extraction conditions.

Extraction variables				Predicted yield (mg/100 g dry powder)	Experimental yield (mg/100 g dry powder)
*X*_1_ (%)	*X*_2_ (°C)	*X*_3_ (h)	*X*_4_ (g/mL)	256	255 ± 2.54 ^a^
55	80	1.2	12		

^a^ Mean ± standard deviation (n = 5).

### 2.2. Static Adsorption Capacity, Adsorption Ratio, Desorption Ratio and Recovery

**Figure 2:**
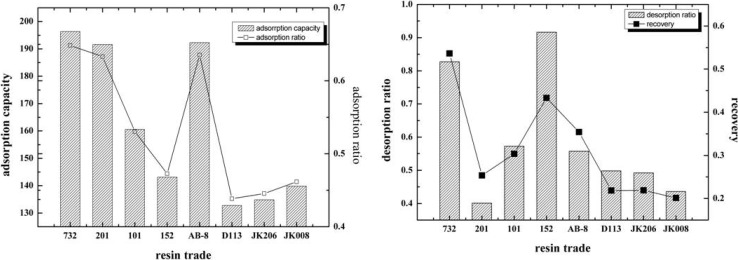
Adsorption capacity, adsorption ratio, desorption ratio and recovery of 1-DNJ with different resins.

The selectivity of resins was based on the capacities of adsorption and desorption, and radio of desorption. The adsorption capacity, adsorption ratio, desorption ratio and recovery of 1-DNJ with different resins were shown in Figure 2. It can be seen that the adsorption capacities of 732, 201 and AB-8 resins were obviously higher than those of other resins. However, the desorption ratio and recovery of 201 resin was the lowest in all resins. While the desorption ratio and recovery of 152 resin was fairly prominent, the adsorption capacity was barely satisfactory. Therefore, 732 and AB-8 resins were selected for the further investigation of adsorption behavior of 1-DNJ from mulberry leaves.

### 2.3. Static Adsorption Isotherms of 1-DNJ with 732 and AB-8 Resins

**Figure 3:**
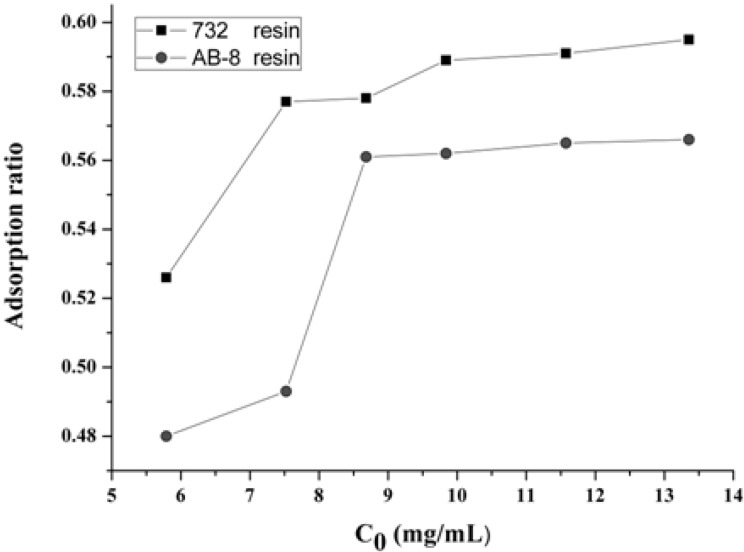
The relationship between the adsorption ratio and the initial concentration of 1-DNJ.

The most suitable initial concentration was achieved by plot between the adsorption ration and the initial concentration of 1-DNJ as shown in Figure 3. It can be seen that the adsorption capacity of 732 and AB-8 resins increased with the initial concentration of 1-DNJ and reached the saturation plateau when the initial concentrations of 1-DNJ were 9.84 and 8.68 mg/mL.

In research on adsorption processes, the Langmuir, Freundlich and Toth isotherms are often used to illustrate the adsorption behavior of solutes in the separation. Adsorption isotherms of 732 and AB-8 resins were conducted at the temperatures of 20, 30 and 40 °C.

**Figure 4:**
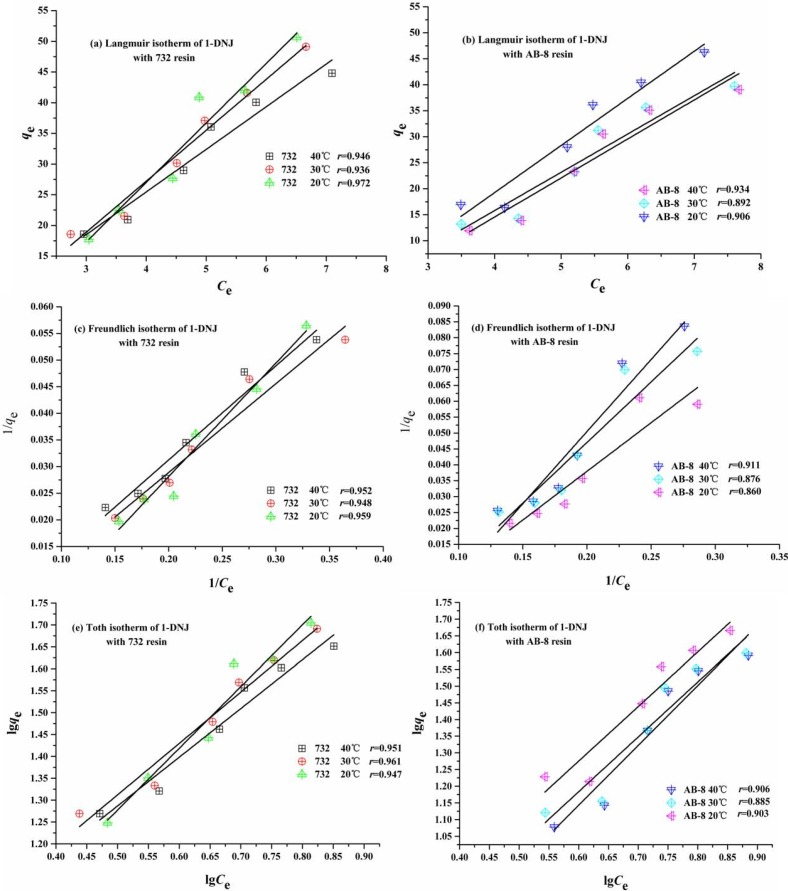
The adsorption isotherms of 1-DNJ with 732 and AB-8 resins at different temperatures.

The isotherms and parameters were obtained from the Langmuir, Freundlich and Toth equations as shown in Figure 4. It can be seen that the correction coefficients of then Langmuir, Freundlich and Toth equations with 732 resin were obviously higher than AB-8 resin. In the comprehensive consideration of the adsorption capacity and desorption ration, 732 resin was selected for the separation of 1-DNJ from mulberry leaves.

Furthermore, the equilibrium adsorption isotherms with 732 resin were highly fitted to the Langmuir models. The Langmuir model assumes monomolecular layer adsorption with the homogeneous distribution of adsorption energies while there is no mutual interaction between adsorbed molecules. The Freundlich model can be used to describe the adsorption behavior of monomolecular layer as well as that of the multimolecular layer [22]. All the adsorption sites on the surface of resin were equivalent and independent, the adsorbates rapidly cover the surface of 732 resin and form one monolayer, the rate during the adsorption process depends on the concentration of the adsorbate in the solution.

### 2.4. Static Adsorption Kinetic of 1-DNJ with 732 Resin

**Figure 5:**
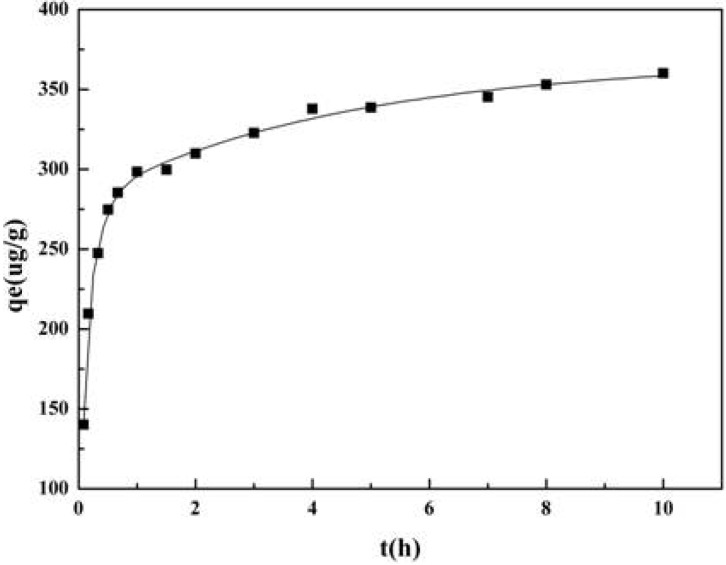
Adsorption kinetic curve of 1-DNJ in mulberry leaves with 732 resin.

In order to reveal the adsorption behavior of 1-DNJ with 732 resin further, the kinetic curve was calculated as shown in Figure 5. It can be seen that the adsorption capacity of 732 resin increased with the increasing adsorption time. In the first two hours, the adsorption capacity increased rapidly and reached the equilibrium at about 4 h.

**Figure 6:**
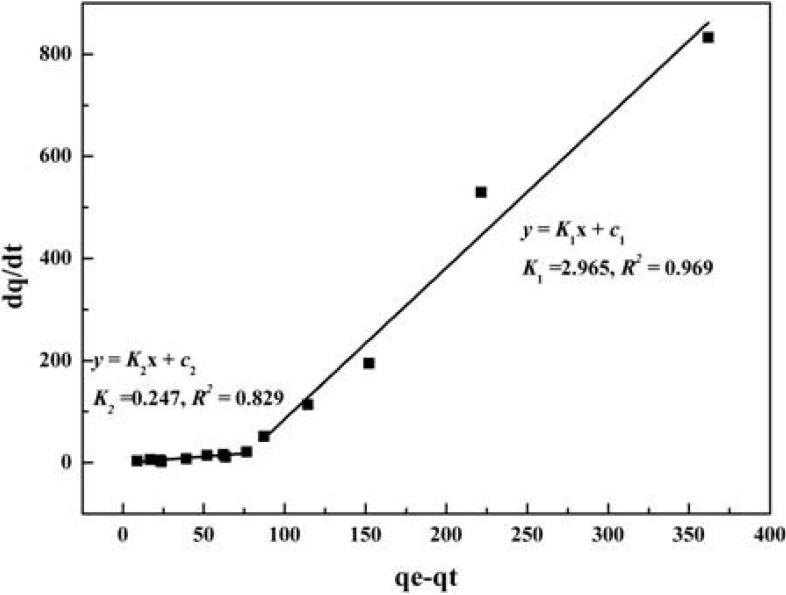
Adsorption rate constants in two-step adsorption kinetic model with 732 resin.

The adsorption process can be seen as the redistribution of the solute molecules between the adsorbents and the liquid phase. Figure 6 shows the adsorption kinetic model which was simulated by the experimental data of the adsorption of 1-DNJ with 732 resin. As can be seen from Figure 6, the adsorption process can be considered as a two-step process. For the two steps, the adsorption rate constants *K*_1_ > *K*_2_, which demonstrates the adsorption process of 1-DNJ with 732 resin may be explained by “an initial fast step followed by a slow step”. In the process of adsorption, the surface coverage by the adsorbed 1-DNJ molecules increases rapidly due to the formation of the first adlayer at the early stage and then trends to gentle by the formation of the second adlayer at the later stage [23].

### 2.5. Dynamic Adsorption Curve of 1-DNI with 732 Resin

**Figure 7:**
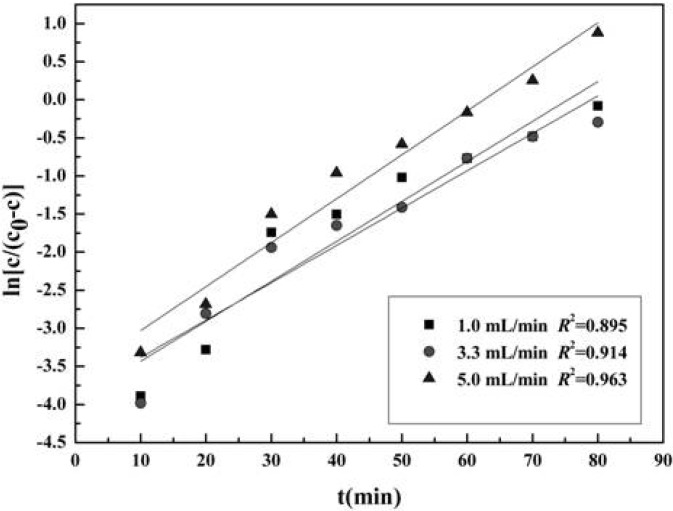
Dynamic adsorption curves of 1-DNJ with 732 resins under different flow rates.

The flow rate of the sample solution is one of the factors influencing the dynamic adsorption. The dynamic adsorption curves of 1-DNJ with 732 resin under different flow rates were obtained based on the linear relationship between ln[*C*/(*C*_0_ − *C*)] and t , as shown in Figure 7. As can be seen from Figure 7, the dynamic leakage time and the adsorption capacity increased as the flow rate increased. In the comprehensive consideration of the dynamic leakage time, adsorption capacity and regression coefficient of adsorption curve, the flow rate of 5 mL/min was selected for the dynamic adsorption of 1-DNJ with 732 resin.

### 2.6. Dynamic Desorption Curve of 1-DNJ with 732 Resin

**Figure 8:**
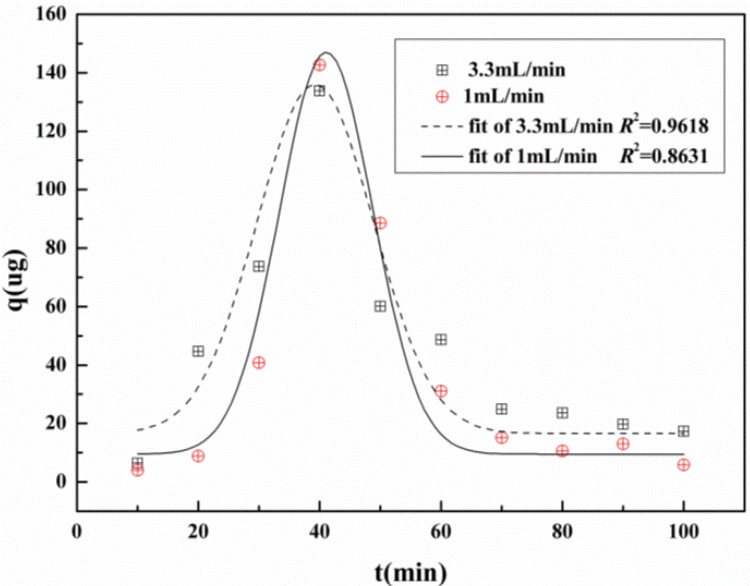
Dynamic desorption curves of 1-DNJ with 732 resins under different flow rates.

The dynamic desorption curves were obtained by plotting the amount of 1-DNJ desorbed and the desorption time. As can be seen from Figure 8, the flow rate is one of the important factors influencing the dynamic desorption curves, and the recovery of 1-DNJ is expected to decrease with increasing flow rate. Meanwhile, it shows that the dynamic desorption curves fitted to the Pearson IV equation as shown in Figure 8. In the comprehensive consideration of the desorption time, recovery and purity of 1-DNJ, the flow rate of desorption solution of 1.0 mL/min was selected as the most suitable flow rate for the dynamic desorption process.

### 2.7. The Results of 1-DNJ Separation with 732 Resin

The preparative separation of 1-DNJ was performed with 732 resin under optimal conditions. Through one treatment with the separation process, a yellowish powder was obtained with a recovery and purity of 1-DNJ in the final product of 90.51% and 15.3%. The results show that separation with 732 resin is a useful method in enriching 1-DNJ from mulberry leaves.

## 3. Experimental

### 3.1. Materials

The 1-DNJ standard was purchased from Tauto Biotech Co., Ltd (Shanghai, China). All other reagents were of analytical grade and deionized water was purified by a Milli-Q water-purification system from Millipore (Bedford, MA, USA). Mulberry leaves were collected from the royal ancient mulberry fields (Daxing District, Beijing, China) in July 2012 and identified by Professor Zhangji of National Institute for Food and Drug Control. The materials were cleaned and dried at 60 °C, then powdered by a FW-100 herb disintegrator (Taisite Equipment Co., Ltd, Tianjin, China) and kept at 4 °C.

Macroporous resins, including AB-8, 201, D101, D152, JK206, JK008, D113 and 732 were purchased from QiRui Petrochemical Company (Shandong, China). The adsorbent beads were pre-treated to remove the monomersand porogenic agents trapped inside the pores during the synthesis process. Prior to use, the resins were soaked into ethanol (95%), shaken for 24 h in room temperature and washed by deionized water thoroughly. The resins were placed into HCl (5%) and the same quantity of NaOH (5%) to remove impurities. Finally, the resins were washed by deionized water until the effluent was chemically neutral [24].

### 3.2. The Extraction and Determination of 1-DNJ

#### 3.2.1. The Extraction of 1-DNJ

The mulberry powder was accurately weighed and refluxed under different extraction conditions (ethanol concentration, extraction temperature, extraction time and ratio of solvent to sample). After the extraction, the extracting solution was filtered and then concentrated under vacuum at 50 °C. The residue was accurately weighted and subjected to HPLC system. The extraction yield was defined as the amount of 1-DNJ in the extracting solution from 100 g dry mulberry powder.

#### 3.2.2. Quantitative Determination of 1-DNJ

The standard solutions of 1-DNJ or the extracted solutions were dissolved with appropriate amount of potassium borate buffer (0.4 M, pH 8.5), and then 40μL FMOC-Cl (5 mmol/L) in CH_3_CN was added. The reactant was mixed immediately and allowed to react at 25 °C for 20 min in a water bath. 10 μL amino acid (0.1 M) was added to terminate the reaction by quenching the remaining FMOC-Cl. The mixture was diluted with 1 mL of 0.1% (*v/v*) aqueous acetic acid to stabilize the DNJ-FMOC, and filtered by a 0.45 μm nylon syringe filter [25,26]. A 10 μL aliquot of the filtrate was injected into the HPLC system. HPLC analysis was carried out on a 2695 Alliance separation module (Waters, Milford, MA, USA) equipped with a Waters temperature control module and a Waters 2475 fluorescence detector (excitation 254 nm, emission 322 nm). A Kromasil C_18_ column (4.6 mm × 250 mm, 5 μm) was used for the analysis. The analyte was eluted with a mobile phase of acetonitrile and 0.1% acetic acid (50:50, *v/v*) with flow rate 1.0 mL/min at 30 °C.

#### 3.2.3. Experimental Designs

RSM was used to find out the optimal extraction conditions for 1-DNJ. The extraction experiments were carried out according to a Box-Behnken design (BBD) with four factors and three levels. The four independent variables selected were ethanol concentration, extraction temperature, extraction time and ratio of solvent to sample as shown in Table 4. For each factor, the experimental range was based on the results of preliminary single-factor experiments. The yield of 1-DNJ extracted from mulberry leaves was the dependent variable. A total of 29 experiments were conducted to optimize the extraction conditions for the extraction procedures [27,28,29,30].

**Table 4:** Levels of the variables of Box-Behnken design (BBD).

Variables	Symbol	Experimental Value		
		Low, -1	Zero, 0	High, 1
Ethanol concentration (%)	*X*_1_	50	60	70
Extraction temperature (°C)	*X*_2_	70	80	90
Extraction time (h)	*X*_3_	1	1.5	2
ratio of solvent to sample (mL/g)	*X*_4_	8	10	12

### 3.3. Static Adsorption and Desorption Tests

The static adsorption tests of 1-DNJ with different resins were performed as follows: 5.0 g of hydrated test resins were placed in flasks and then 25 mL of sample solutions of mulberry leaves extracts (the concentration of 1-DNJ 6.95 mg/mL) were added. The flasks were shaken (100 rpm) for 24 h at 25 °C. The solutions after adsorption were analyzed by chromatography. The desorption processes were performed as follows: after reaching adsorption equilibrium, the resins were first washed by deionized water and then desorbed with 20 mL Ammonia (1.0 mol/mL). The flasks were shaken (100 rpm) for 24 h at 25 °C, then the desorption solutions were analyzed by chromatography.

### 3.4. Static Adsorption Equilibrium Isotherms Tests

The tests for equilibrium adsorption isotherms [31,32,33,34] with 732 and AB-8 resins were conducted by mixing 30 mL sample solutions of mulberry leaves extracts (the concentration of 1-DNJ 6.95 mg/mL) at different concentrations with 5 g (dry weight) resins, and then shaking for 24 h at the temperature of 20 °C, 30 °C and 40 °C. The initial and equilibrium concentrations at different temperatures were determined by chromatography.

The Langmuir isotherm theory is based on the assumption of adsorption on a homogeneous surface. The equation can be described in the following form:

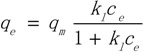
(3)
where *q_e_* is the equilibrium amount of adsorbent (µg/g), *c_e_* is the equilibrium concentration. The Freundlich equation is also used to describe the adsorption process. It can be written in the form:

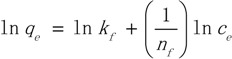
(4)

The Toth model is applied to adsorption on heterogenerous surfaces. This equation is written in the following form:

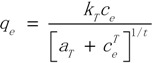
(5)

### 3.5. Static Adsorption Kinetic Tests

The tests for adsorption kinetic [35,36] with 732 resin were conducted by mixing 50 mL sample solutions of mulberry leaves extracts (the concentration of 1-DNJ 6.95 mg/mL) with 5.0 g (dry weight) resins, then shaken (100 rpm) for 10 h at a temperature of 25 °C. The solutions were analyzed by chromatography at certain time till equilibrium.

The experimental data of adsorption rate constant were fitted to the two-step adsorption kinetics model to describe the adsorption kinetic property of solutions with the resin:

*q_t_* = *q_e_* − *A*_1_*e*^−*k*_1_*t*^ − *A*_2_*e*^−*k*_2_*t*^ (6)

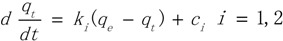
(7)
where *A*_1_ and *A*_2_ are frequency factors; *k*_1_ and *k*_2_ are adsorption rate constants; *q_t_* is the adsorption quantity (mg/g) [37].

### 3.6. Dynamic Adsorption and Desorption Tests

The dynamic adsorption and desorption tests were performed on glass (25 × 500 mm) columns with a known dosage of 732 resins. Sample solution flowed through the glass columns at the rate of 5 mL/min. The concentration of 1-DNJ in the effluent liquid was analyzed by chromatography at certain time. After reaching adsorption equilibrium, the adsorbed column was washed by deionized water firstly, and then eluted by ammonia (1 mol/mL) at 1 mL/min. The concentration of 1-DNJ in desorption solution was determined by chromatography.

### 3.7. The Preparative Separation of 1-DNJ with 732 Resin under Optimal Conditions

The preparative separation of 1-DNJ was performed with 732 resin under optimal conditions. Sample solution was flowed through the glass columns at the rate of 5 mL/min with the concentration of 9.84 mg/mL. After reaching adsorption equilibrium, the adsorbed column was washed with four column volumes of deionized water and four column volumes of 10% ethanol. Ammonia (1 mol/L) was selected to elute at the rate of 1 mL/min and the eluent was concentrated under vacuum. The concentrated solution was diluted to a certain volume with deionized water and then spray drying to give the finally product. The product was weighed and subjected to chromatography.

## 4. Conclusions

The extraction and separation method of 1-DNJ from mulberry leaves with resins was successfully demonstrated in this study. The maximal yield of 1-DNJ was obtained from mulberry leaves when they were extracted under conditions of 55% ethanol concentration, extraction temperature of 80 °C, extraction time of 1.2 h and ratio of solvent to sample of 12:1. Among the eight resins that were investigated, 732 resin offers the best separation capacity for 1-DNJ from other components in mulberry leaves extracts. Process parameters, including concentration and flow rate of sample solution, concentration and flow rate of desorption solution at different temperatures were optimized for the most effective separation of 1-DNJ from mulberry leaves with 732 resin. The equilibrium experimental data of adsorption of 1-DNJ from mulberry leaves with 732 resin at different temperatures fitted to Langmuir isotherms. The adsorption kinetics process for 1-DNJ in mulberry leaves with 732 resin fitted to two-step adsorption kinetics model. Through one run treatment on the column packed with 732 resin under optimal conditions, the recovery and purity of 1-DNJ in the final product was 90.51% and 15.3%.
